# Prevalence and significance of psammoma bodies in cervicovaginal smears in a cervical cancer screening program with emphasis on a case of primary bilateral ovarian psammocarcinoma

**DOI:** 10.1186/1742-6413-5-7

**Published:** 2008-04-16

**Authors:** Teresa Pusiol, Anna M Parolari, Irene Piscioli, Luca Morelli, Franca Del Nonno, Stefano Licci

**Affiliations:** 1 Department of Pathology, "S. Maria del Carmine" Hospital, Rovereto (TN), Italy; 2 Department of Gynecology, New Hospital of Arco (TN), Italy; 3 Department of Radiology, Civil Hospital of Budrio (BO), Italy; 4 Department of Pathology, "National Institute for Infectious Diseases – L. Spallanzani" IRCCS, Rome, Italy

## Abstract

**Background:**

The purpose of our study was to determine the prevalence and significance of psammoma bodies (PBs) in the cervicovaginal smears of the screening population of Trento district (Italy), with the description of the cytological presentation of an asymptomatic bilateral ovarian psammocarcinoma.

**Methods:**

From 1993 to 2006, women with PBs detected on consecutively screened cervical smears were identified from the computerized pathology database of Rovereto Hospital. The follow-up period was set from the time of cytological diagnosis to May 31^st^, 2007. Clinical information was obtained from retrospective review of women's medical records. The source of PBs was identified with adequate diagnostic procedures.

**Results:**

PBs were found in six of the 201,231 Papanicolaou screening smears (0.0029%). Benign conditions (intrauterine device, inclusion ovarian cysts and ovarian cystoadenofibroma with PBs) were found in four patients. In two cases, PBs were associated with malignant cells; a bilateral ovarian malignancy was diagnosed in both cases, a serous adenocarcinoma and a psammocarcinoma.

**Conclusion:**

PBs in the cervicovaginal smears are a rare finding, associated more often with benign conditions than with malignancies. Moreover, to our knowledge, our case of primary ovarian psammocarcinoma is the first report in which the presence of malignant cells and PBs in the cervicovaginal and endometrial smears represents the first manifestation of disease.

## Background

The presence of psammoma bodies (PBs) in cervicovaginal smears (CVS) is a rare finding, the prevalence ranging from 0.00047% to 0.057% in large series studies [1-6]. PBs on Papanicolau smears have been described in association with a wide variety of gynaecological conditions including malignant tumors, such as malignant serous epithelial ovarian tumors [7], endometrial carcinoma with PBs [8], neuroendocrine carcinoma of the cervix [9], primary peritoneal borderline tumour [10], tubal serous carcinoma and cervical clear cell carcinoma [2]. Exceptionally, the primary site of origin remains unclear [1,4]. However, they are most frequently associated with benign conditions, presumably related to the use of an intrauterine device (IUD) [11-13], to a history of oral contraception [14] or associated with a benign ovarian neoplasm [15], inflammatory lesions, such as tubo-ovarian adhesions and endosalpingiosis [16,17], endometrial benign hyperplasia and endometrial polyps [3]. The most reliable predictor of a malignancy is the association of malignant cells with PBs on the smears. The purpose of our work is to determine the prevalence and significance of PBs in a sequential series of CVS in the screening population of Trento district (Italy). Furthermore, we illustrate the first case report, as far as we are aware, of a bilateral primary ovarian psammocarcinoma (PC), in which PBs found in association with malignant cells in the cervicovaginal and endometrial smears represent the first manifestation of disease.

## Methods

We identified from the computerized pathology database of Rovereto Hospital all cases describing the presence of PBs in CVS, with a retrospective review of 201,231 screening cytological exams from 1993 to 2006. The cervical cancer screening program was performed according to Walton report [18] and cytological diagnoses were reported according to the Bethesda System and its modifications in the years [19,20]. The diagnostic connotations were recorded using standardized Systematized Nomenclature of Medicine coding (PBs: M-55580). Clinical informations were obtained from retrospective review of women's medical records before and after identification of PBs (age, gravity, parity, menopausal status, symptoms present at the time of abnormal papanicolau smear, contraceptive and gynaecological history). Follow up informations were collected from the time of cytological diagnosis to May 31^st^, 2007. Adequate diagnostic procedures (endometrial aspiration, hysteroscopy, uterine curettage, vaginal and abdominal ecography, TC) were performed in order to identify the source of PBs.

## Results and Discussion

In the retrospective review of 201,231 screening program cytology reports we identified 6 Papanicolaou smears with PBs (0.0029%).

**Figure 1:**
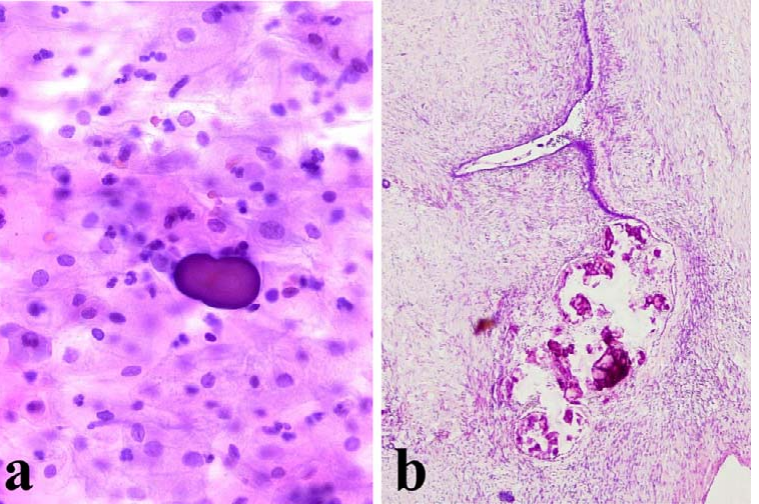
**a)** Presence of a psammoma body in absence of atypical cells in cervicovaginal smear (Papanicolaou stain, 200×); **b) **Serous ovarian cystoadenofibroma with parietal psammoma bodies (Hematoxylin and Eosin, 100×).

Four of them belonged to premenopausal women and PBs were found in absence of atypical cells (Fig 1a). Endometrial aspiration was negative either for PBs or for malignant cells.

Two of these patients (40 years old, gravida 2 para 2 and 36 years old, gravida 1 para 1) had an IUD. Because of a possible coexistence of a gynaecologic malignancy the surgeons performed a laparoscopy with removal of the IUD. The abdominal and pelvic organs, including the ovaries, revealed no abnormalities.

The third patient (46 years old, gravida 1 para 2) showed multiple, small cysts in the right ovary. A laparoscopic resection was performed and histological examination revealed benign inclusion ovarian cysts with intraparietal PBs.

In the fourth patient (45 years old, gravida 1, para 1), a pelvic ultrasonography showed a cystic structure, 1.5 cm in diameter, in the right ovary. For not specified circumstances, an abdominal hysterectomy with bilateral salpingo-oophorectomy and appendicectomy was performed. Histopathologic examination revealed a serous cystoadenofibroma with PBs of the right ovary (Fig 1b). The left ovary had no significant lesions.

Papanicolau smears were repeated three to six months after the initial identification of PBs in the four patients, and none of them showed persistent PBs in the smears, even after a follow up period of 3–8 years.

**Figure 2:**
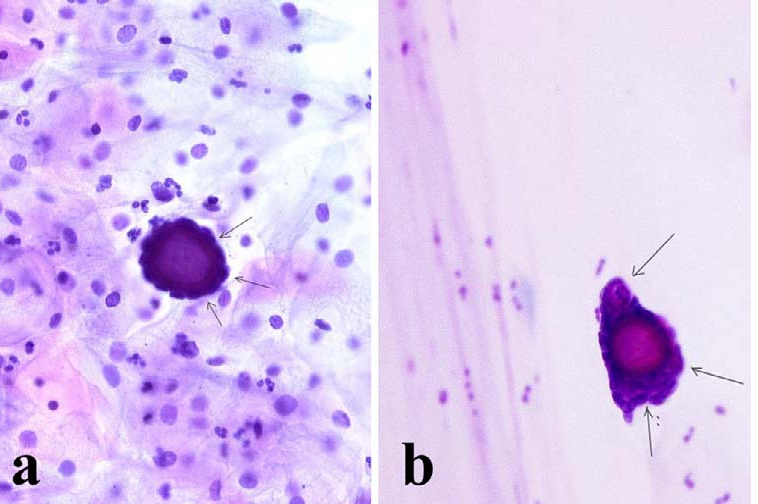
**a) **Psammoma body surrounded by atypical cells (arrows) in cervicovaginal smear (Papanicolaou stain, 200×); **b) **Psammoma body surrounded by atypical cells (arrows) in endometrial aspiration smear (Papanicolaou stain, 200×).

The last two patients with PBs on the smears had a bilateral ovarian malignancy with a diffuse involvement of the abdominal cavity. Rare blue-stained, laminated, calcified bodies, identified as PBs were found in association with diagnostic malignant cells, which were variable in size and shape and had large nuclei with prominent nucleoli (Fig 2a). Both endometrial aspirations contained several PBs isolated or surrounded by clusters of columnar cells showing a moderate degree of atypia (Fig 2b). Rare papillary groups of atypical glandular cells were present. Both patients underwent an exploratory laparotomy, with a subsequent total abdominal hysterectomy, bilateral salpingo-oophorectomy, omentectomy, regional lymphadenectomy and multiple peritoneal biopsies.

**Figure 3:**
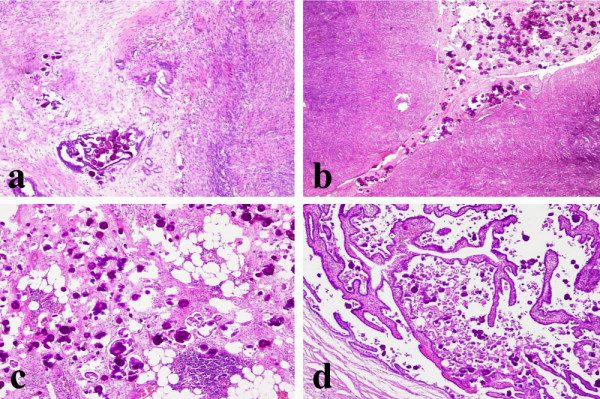
**a) **Moderately differentiated ovarian adenocarcinoma with psammoma bodies infiltrating the stroma (Hematoxylin and Eosin, 100×); **b) **Psammocarcinoma localized on the ovarian surface with invasion of the underlying stroma (Hematoxylin and Eosin, 100×); **c) **Omental metastasis of psammocarcinoma (Hematoxylin and Eosin, 100×); **d) **Presence of psammocarcinoma in the tubaric lumen (Hematoxylin and Eosin, 100×).

In one patient (56-years-old, gravida 2, para 2) a preoperative vaginal ultrasonography revealed a dishomogeneous uterine corpus, 7,3 × 5,4 cm in size, with multiple nodular lesions referable to leiomyomas located in the anterior wall of the myometrium. Intraoperatory findings showed a right ovarian mass, white and firm, 4 cm in diameter and another nodular lesion on the surface of the left ovary, grey-white, well circumscribed and 2 cm in diameter. The invasive thickness of the ovarian stroma was 1,2 cm on the cut surface. The omentum and peritoneal surface were involved by multiple tumor nodules (2 cm in maximum diameter). Intraoperative histological examination was consistent with the diagnosis of bilateral moderately differentiated ovarian serous adenocarcinoma. The final pathologic report confirmed the diagnosis made on frozen sections. Additonal feature was the presence of multiple psammomatous calcifications (Fig 3a). Neoplastic infiltration was observed in the endometrium, without myometrium invasion. The omental nodules showed the features of invasive implants. Malignant cells and PBs were found in the peritoneal washing. Twenty-seven regional lymph nodes were free of malignancy. She was placed in the International Federation of Gynecology and Obstetrics (FIGO) stage III B. The patient received chemotherapy. Since then, she has been followed up with several CT scans of abdomen and pelvis and has recovered without incident.

In the other patient (50 years-old, gravida 1, para 1) exploratory laparotomy showed a diffuse white-grey nodularity on the surface of both ovaries. Two omental and three peritoneal nodules (2 cm in maximum diameter) were found. Intraoperative histological examination was consistent with the diagnosis of bilateral ovarian serous carcinoma with extensive psammoma bodies formation, showing the typical proliferative pattern of PC, according to the diagnostic criteria defined by Gilks [21], and infiltrating the ovarian stroma (invasive tickness > 1 cm) (Fig 3b). The nodules removed from the peritoneal and omental surfaces were confirmed to be neoplastic involvement (Fig 3c). The final histological diagnosis was primary bilateral psammocarcinoma of the ovaries with macroscopic omental and peritoneal metastases, invasion of both tubaric lumens (Fig 3d), extension to endometrium without myometrium infiltration. The eighteen lymph nodes sampled were free of disease. She was placed in the FIGO stage III B. Following surgery no gross tumor was remaining. The peritoneal cytology was positive. The patient was started on chemotherapy. After 10.5 years of follow up the patient is alive, free of disease.

The prevalence of PBs in our series of consecutively screened Papanicolau smears is 6 per 201,231 (0.0029%), which confirms that this is a very unusual finding, in agreement with other large series of CVS previously reported in the literature and summarized in Table 1. The actual prevalence and significance of PBs in CVS in a cervical cancer screening program, based on the Walton report [18], have not been previously reported. The Walton report, a document of the Canadian task force on cervical cancer screening, recommended a program based on chronologic age, calling for 3- and 5-year intervals between examinations. The six cases described in our study exhibit different clinical diagnoses, with a higher prevalence of benign lesions associated with PBs, according to the literature data.

**Table 1:** Prevalence of psammoma bodies in cervicovaginal smears in large series studies

**Authors**	**Number of cases**	**PBs in smears**	**Prevalence (%)**
Nicklin et al.	4,685,454	22	0,00047
Parkash et al.	82,480	8	0,0096
Muntz et al.	400,255	25	0,0062
Kern et al.	234,318	7	0,0030
De Peralta-Venturina et al.	421,358	24	0,057
Zreick et al.	34,816	18	0,052
Our study	201,231	6	0,0029

**Tot.**	**6,059,912**	**110**	**0,0018**

The observation of PBs on CVS should not be ignored and should alert the clinician in order to evaluate the possibility of a coexistent carcinoma, even though a review of the literature reveals that in more than 50% of patients PBs are associated with benign conditions [1-6]. All benign cases are described in relatively young individuals, the oldest being 46 years old. In two of our cases the presence of IUD was considered the source of PBs on the basis of similar, well documented reported cases [11-13]. Another source of PBs in cervicovaginal smears are benign inclusion cysts [22], like in another case of ours. The rupture of an ovarian cyst, followed by aspiration of its contents and of minute parietal fragments with PBs by the fallopian tube is the most likely explanation for the cytological finding. Calcifications and PBs are present in almost the 30% of ovarian cystoadenofibromas [23] and this can justify the presence of PBs in CVS, like in the fourth benign case of ours; the same mechanism above described may be evoked.

When PBs in the CVS are associated with malignant or borderline tumors, the ovary is the most common primary site (51%), serous cystoadenocarcinoma being the most common neoplasm, followed by the uterine corpus (30%) [24]. In our two cases, the presence of malignant cells with PBs in the lumen of the fallopian tubes may be the mechanism responsible for the cytological findings. In one of them, the report was related to an unknown primary bilateral ovarian PC. This represents a rare invasive variant of serous carcinoma arising in the ovary and in the peritoneum [21,25-31].

In the first description by Gilks et al. [21], eight cases were primary ovarian neoplasms and three were arising from omentum. These authors reported the accurate strict histological diagnostic criteria for the diagnosis of PC, according to which psammoma bodies must replace at least 75% of the papillae, cytological features must be low grade and the neoplastic epithelium must be arranged in small nests with no areas of solid proliferation. Potential mechanisms responsible for the characteristic, extensive PBs formation include the accumulation of successive layers of calcium on single necrotic or degenerated tumor cells [32].

Primary peritoneal PC is a very unusual variant of extraovarian neoplasms that histologically resemble surface-epithelial-stromal tumours of ovarian origin.

In these cases, an adequate sampling of the omentum, the adnexa, and the uterine serosa, as well as of any dominant mass, is required to establish the primary origin of the malignancy.

In our case, a stromal invasion with tumor size more than 5 × 5 mm in diameter and the extensive involvement of the ovary surface have been considered the conclusive criteria of primary bilateral ovarian PC.

Clinical behaviour of PCs closely resembles that of borderline serous tumours with a protracted course and a favourable prognosis. Nevertheless, tumors with an aggressive course have been described in the literature [30,31]. In the case reported by Poggi et al. [30] no adjuvant therapy was proposed because of the supposed indolent behaviour, with a recurrence of disease after eighteen months. In the second case described by Akbulut et al. [31], 9 cycles of chemotherapy were performed, after a complete debulking, with a recurrence of the disease after 5 years and metastatic infiltration of the vertebral corpus one year later, in spite of other 6 cycles of chemotherapy.

Our patient chose to receive adjuvant chemotherapy and is alive and free from disease after 10.5 years of follow-up. The excellent prognosis is in agreement with previous reports.

## Conclusion

Our study performed on a large screening series confirms that the presence of PBs in CVS is a rare finding, more often associated with benign conditions. Anyway, this finding should never be underestimated and a careful examination of the morphological features of the cell groups is crucial to establish the source of PBs and disclose an underlying malignancy. Additional clinical investigations are always recommendable.

Moreover, we have described the first report of ovarian PC in which the presence of malignant cells and PBs in the cervicovaginal and endometrial smears represents the first manifestation of the disease.

## Competing interests

The authors declare that they have no competing interests.

## Authors' contributions

TP, AMP, IP and LM participated equally in the design of the paper and in the collection of the clinico-pathological data. FDN and SL participated in the revaluation of the cytological and histological specimens and in the drafting of the manuscript. SL revised critically the final version of the manuscript. All authors read and approved the final manuscript.
